# Supplementary material for: Pan-Cancer Analysis Reveals AEBP1-Collagen Co-Expression and Its Potential Role in CAF-Mediated Tumor Stiffness
Source: Int J Mol Sci. 2025 Nov 27;26(23):11474. doi: 10.3390/ijms262311474 (PMC12691837; doi:10.3390/ijms262311474)
Supplement: Supplementary file 1 [file ijms-26-11474-s001.zip › ijms-3966965-supplementary.pdf]

Supplementary Figure S1

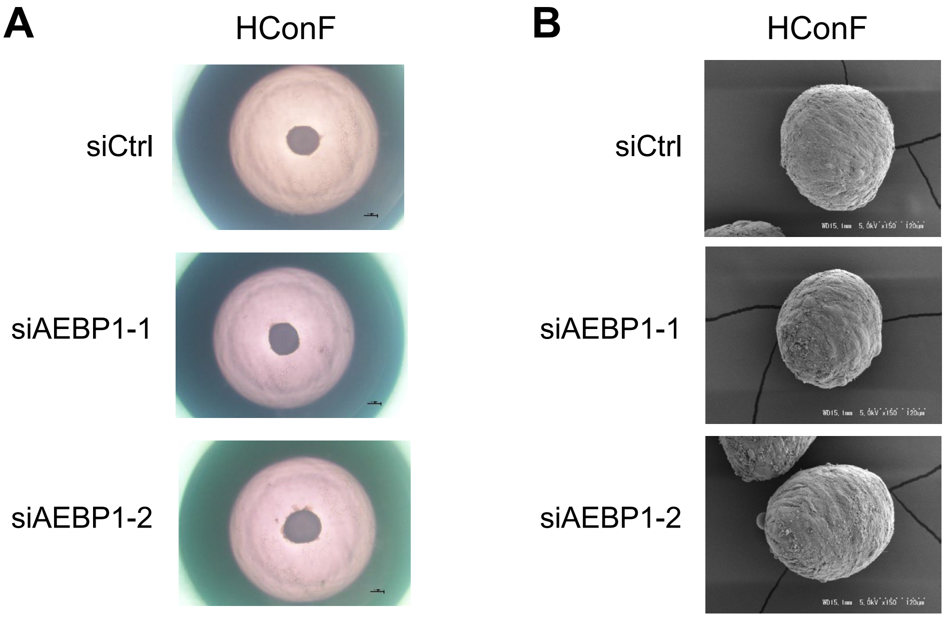

AEBP1 knockdown did not significantly affect size and morphology of 3D spheroids derived from normal fibroblasts. (A) Representative phase-contrast micrographs of 3D spheroids derived from HconF cells transfected with a control siRNA or an siRNA targeting AEBP1 (siAEBP1-1 or siAEBP1 2). (B) Representative scanning electron micrographs of 3D spheroids derived from HconF transfected with an indicated siRNAs.
